# Simulated Respiratory Secretion for Use in the Development of Influenza Diagnostic Assays

**DOI:** 10.1371/journal.pone.0166800

**Published:** 2016-11-21

**Authors:** Michael E. Bose, Kate C. McCaul, Hong Mei, Amy Sasman, Jie He, William Kramp, Roxanne Shively, Ke Yan, Kelly J. Henrickson

**Affiliations:** 1 Midwest Respiratory Virus Program, Department of Pediatrics, Medical College of Wisconsin, Milwaukee, Wisconsin, United States of America; 2 Biomedical Advanced Research and Development Authority, Office of the Assistant Secretary for Preparedness and Response, US Department of Health and Human Services, Washington, DC, United States of America; 3 Section of Quantitative Health Sciences, Department of Pediatrics, Medical College of Wisconsin, Milwaukee, Wisconsin, United States of America; University of Hong Kong, HONG KONG

## Abstract

Many assays have been developed for the detection of influenza virus which is an important respiratory pathogen. Development of these assays commonly involves the use of human clinical samples for validation of their performance. However, clinical samples can be difficult to obtain, deteriorate over time, and be inconsistent in composition. The goal of this study was to develop a simulated respiratory secretion (SRS) that could act as a surrogate for clinical samples. To this end, we determined the effects major respiratory secretion components (Na^+^, K^+^, Ca^2+^, cells, albumin IgG, IgM, and mucin) have on the performance of influenza assays including both nucleic acid amplification and rapid antigen assays. Minimal effects on the molecular assays were observed for all of the components tested, except for serum derived human IgG, which suppressed the signal of the rapid antigen assays. Using dot blots we were able to show anti-influenza nucleoprotein IgG antibodies are common in human respiratory samples. We composed a SRS that contained mid-point levels of human respiratory sample components and studied its effect compared to phosphate buffered saline and virus negative clinical sample matrix on the Veritor, Sofia, CDC RT-PCR, Simplexa, cobas Liat, and Alere i influenza assays. Our results demonstrated that a SRS can interact with a variety of test methods in a similar manner to clinical samples with a similar impact on test performance.

## Introduction

Influenza is an important respiratory virus that infects millions of people each year and can lead to severe illness and hundreds of thousands of deaths worldwide. Because of its prevalence and potential for severe illness, there have been many diagnostic assays developed for the detection of influenza viruses. These methodologies include: detection of influenza virus proteins using immunoassays (e.g., rapid antigen tests (RATs)) or nucleic acid amplification tests (NAAT) (e.g., real-time RT-PCR), or the decreasingly common traditional methods of viral tissue culture and direct fluorescent microscopy. Additionally, more rapid methods of virus detection are trending toward use of respiratory tract swab specimens that are tested directly without dilution and stabilization in viral transport media. During the development of these assays, analytical studies were commonly used to assess virus detection in a background matrix prior to evaluating detection in clinical samples. Assay developers have traditionally used archived, leftover, de-identified respiratory samples that were often pooled. However, the availability of these samples may be limited and may not represent the general population. Additionally, there are increasing concerns with genetic information contained in such samples thereby leading to increased regulations regarding retention of clinical samples. Also, results may not be reproducible due to large variability in clinical sample composition, specimen collection, and/or storage methods. Thus, clinical samples are not necessarily ideal for development purposes. An artificial matrix (i.e., simulated respiratory secretion (SRS)) that reflects the biological, chemical, and physical characteristics of respiratory secretions could be useful for developers with limited availability to suitable clinical samples.

Human respiratory secretions, typically collected as the substrate for influenza virus detection, are a complex matrix containing a variety of host components in addition to an infecting virus and commensals. Even though a number of studies report investigating the concentrations of these components [1–7], it is generally not well understood how these components interact to affect the reactivity with different diagnostic assay methods. In this study, the effects of major respiratory sample components on representative influenza diagnostic assays were evaluated, and an SRS formulated that could be used as a matrix during development of influenza diagnostics assays.

## Materials and Methods

### Ethics statement

This study was approved by the Medical College of Wisconsin Institutional Review Board and allows for the collection of de-identified samples from various institutions.

### Quantification of respiratory sample components in nasopharyngeal swab (NPS) specimens stored in viral transport media (VTM)

De-identified NPS specimens were collected from Children’s Hospital of Wisconsin (CHW) and Dynacare laboratories. Ten samples from children (< 18 years old) were collected at CHW and ten samples from adults (≥ 18 years old) at Dynacare. The CHW samples were collected and stored in 1.5 ml of M6 transport media (Remel, Lenexa, KS, USA). The samples from Dynacare were collected and stored in 3.0 ml of M6 transport media (Remel). Samples were remnants from routine clinical testing for influenza A and B and Respiratory Syncytial Virus (RSV) and were collected between January 31, 2015 and April 30, 2015 and stored at -80°C. After transport to our lab, the samples were thawed and aliquoted into appropriate volumes for each of the quantification assays. Aliquots were stored at -80°C until use.

The quantity of albumin, IgG, IgA, IgM, and nucleic acid were determined. Albumin, IgG, IgA, and IgM were quantified using ELISAs (Model # KA0455, KA3817, KA1855, and KA2110, Abnova Corporation, Zhongli District, Taiwan). Assays were performed as described in the product manual following the instructions for saliva with samples diluted 1:500 for the albumin and IgA assays, 1:1000 for the IgG assay, and 1:100 for the IgM assay. Several samples needed to be diluted 1:5000 for the albumin and IgA assays to fall within the range of the standards. Each of these assays comes with a standard that is 2-fold serially diluted to generate a standard curve for each run. Data were analyzed using a four parameter regression for the standard curves on the MyAssays.com website (http://www.myassays.com/). Nucleic acid quantity was determined by extraction with an easyMAG and quantification with a Nanodrop 2000c (Thermo Fisher Scientific, Waltham, MA, USA). The concentrations were determined in triplicate for each component for each sample.

Levels of components from children and adults were compared using a generalized linear model with repeated measures for albumin, IgA, IgG, IgM, and nucleic acid, which had three replicates. Compound symmetry was used as the correlation matrix for the measurements within subjects. No data sets had a normal distribution. All of the components except albumin were log base 2 transformed. Albumin, which was skewed by a few exceptionally high values, was transformed using 1 over the square root of the original value. Also, correlations between each of the components were determined using Spearman’s rank correlations.

### Components for effects model testing and preparing SRS

Concentration ranges for major compositional components of respiratory secretions used in these studies were compiled from cited references (Table 1).

**Table 1:** Simulated respiratory secretion components and ranges.

Component	Range	References	Source^a^
Na^+^	150–211 mM	[4,7]	NaCl, BDH, 0241
K^+^	4.1–16.6 mM	[4,6,7]	KCl, BDH, 0395-VBD
Ca^++^	2.5–8.1 mM	[4,6,7]	CaCl_2_, Spectrum, C1096
Epithelial Cells	1×10^6^–1×10^7^ cells/ml	[5]	A549, ATCC, CCL-185
Albumin	0.23–1.05 mg/ml	[3,4]	Human Albumin, Sigma, A1653
IgG	0.051–1.36 mg/ml	[1,3,4,6]	Human IgG, Sigma, 56834
IgM	0.014–0.158 mg/ml	[1]	Human IgM, Millipore, AG722^b^
Polysaccharide / Mucin	1.0–4.22 mg/ml	[6]	Bovine Mucin, Sigma, M3895

^a^ Lists the item being used, the manufacturer, and the model number.

^b^ Isolated from serum IgM myeloma patients. This was used only for the fractional factorial design experiment. Human IgM purified from normal healthy patients was used for remaining studies (Sigma, I8260).

### Viruses

Viruses were provided by the Influenza Division, WHO Collaborating Center for Surveillance, Epidemiology and Control of Influenza, Centers for Disease Control and Prevention (CDC), Atlanta, GA, USA. The five influenza viruses used in this study were A/Minnesota/03/2011 (H1N1pdm), A/Montana/05/2011 (H3N2), A/Indiana/10/2011 (H3N2v), B/Nevada/03/2011 (Victoria-like), and B/Wisconsin/01/2010 (Yamagata-like). Viruses were propagated in MDCK cells (Model#CCL-185, obtained directly from ATCC, Manassas, VA, USA) and quantified by TCID_50_ using the Reed-Muench method [8]. Viruses were stored at -80°C until thawed on day of use, and diluted in 1X PBS (Phosphate Buffered Saline) (Model# D8537, Sigma-Aldrich, St. Louis, MO, USA) when appropriate.

### Influenza assays

The assays used included the BD Veritor Flu A+B Test—For Swab Specimens (Model#256045, Becton, Dickinson and Company, Franklin Lakes, NJ, USA), the Sofia Influenza A+B FIA (Model#20218, Quidel, San Diego, CA, USA), the CDC Influenza A, Influenza B, RNaseP Real-Time RT-PCR Assay (Model#FR-198, Influenza Reagent Resource, Manassas, VA, USA), the Simplexa Flu A/B & RSV Direct assay (Model#MOL2650, Focus Diagnostics, Cypress, CA, USA), the cobas Liat Influenza A/B assay (Model# 07341890190, Roche, Basel, Switzerland), the Alere i Influenza A & B assay (Model#425–000, Alere, Waltham, MA, USA), and a virus culture method in MDCK cells (Model#CCL-34, ATCC, Manassas, VA, USA) with TCID_50_ for quantification. The Alere i and cobas Liat assays were not released at the beginning of this work and were included in only some of the experiments. Becton, Dickinson and Company and Quidel provided research software which allowed viewing of the raw signal data from the Veritor and Sofia instruments and which facilitated analysis of these assays.

### Infected cells

A549 cells (Model#CCL-34, obtained directly from ATCC) were grown until 80% to 90% confluence and then inoculated with influenza virus. At the first sign of cytopathic effect (8 to 10 h), cells were harvested. Cells were washed twice with PBS to remove cell free virus, re-suspended, and then stored at -80°C until used. The number of cells was counted microscopically with trypan blue staining and a hemocytometer. The percentage of infected cells was determined with the Light Diagnostics Influenza A & B DFA (EMD Millipore, Billerica, MA, USA). The viral RNA within infected cell stocks was approximated by a quantitative real-time RT-PCR.

### Effect of respiratory secretion components on influenza assays

To identify the appropriate dilutions for each test/virus, an initial screening with each virus and test was performed with ten-fold serial dilutions. Dilutions were selected that were approximately 1 log above the limit of detection for each virus/test when possible (S1 Table). For this study, a 2^7−4^ resolution three fractional factorial design (using the high and low limits listed in Table 1) was selected (S2 Table) resulting in eight formulations that allowed for the separation of main effects. The main effects were confounded with two-factor interactions due to the compactness of this design.

Dilutions of the five influenza viruses in each of the component formulations were tested with the Sofia, Veritor, Simplexa, CDC RT-PCR, and virus culture assays. A single dilution of each virus was tested with three replicates at that dilution. All eight formulations were tested per day with one virus and all assays. Virus dilutions were prepared in freshly made component formulations and stored at 4°C or on ice until used. For each test, 50 μl of sample was absorbed on a flocked swab (Model # 503CS01, Copan Diagnostics, Murrieta, CA, USA) and then tested in the assay either directly for the Sofia and Veritor assays or placed in 3 ml UniTranz-RT Transport Media (Puritan Medical, Guilford, ME, USA) and then tested with the CDC, Simplexa, and virus culture assays. For the CDC assay, easyMAG extraction (bioMerieux, Marcy-l’Etoile, France) was performed before amplification on the ABI 7500 (Thermo Fisher Scientific, Waltham, MA, USA).

The component fractional factorial data was analyzed using a linear regression model in which each of the components was considered a variable using the R statistical software version 3.1.1 (R Foundation for Statistical Computing, Vienna, Austria). The mean value for each formulation was calculated for each virus and the viruses acted as repeats. A log_2_ transformation was performed on the Sofia and Veritor data prior to analysis. The data for Sofia and Veritor assays also was analyzed with a random effects model where the IgG effect was allowed to vary across viruses.

An extension of this testing was done with the Liat assay (not available during the original respiratory component screening experiment). H1N1pdm was diluted to a 10^−4^ dilution from stock in SRS, PBS, and PBS supplemented individually with cations (Na+, K+, and Ca++), albumin, cells, IgG, IgM, or mucin. The concentration for each component was the midpoint of the range in Table 1. Each component was compared to the PBS only data and significance (p-values) calculated using a two-sample t-test assuming equal variances.

### Assessment of matrix effects using SRS, PBS, and pooled negative clinical sample (NCS)

Virus dilutions selected for this study (S3 Table) were based on a preliminary screening experiment. NCS was prepared from nasopharyngeal swab specimens that were self-collected voluntarily by three adult lab members and placed in 3 ml of UniTranz-RT Transport Media (Puritan Medical). Each person collected one swab per nostril for five days. Specimens from each day were pooled and screened for influenza using the CDC assay. All pools were negative. All NCS was pooled, aliquoted into single use aliquots, and stored at -80°C until use. The SRS formulation used contained the midpoint concentration of the range listed for each component in Table 1 and was prepared on the day of testing.

On the day of testing, one virus was diluted in PBS and the final dilutions for each test were made in the appropriate matrix (PBS, SRS, or NCS). Dilutions were made into three 50 μl aliquots. Then, each aliquot was absorbed on a swab and tested in each of the assays. Virus, matrix, and dilution order were randomized prior to testing. For the CDC, Liat, and Simplexa assays the NCS swab was placed into a 3 ml aliquot of NCS rather than just transport media so as to maintain the appropriate concentrations of respiratory sample components.

Data for the CDC, Liat, Simplexa, Sofia, and Veritor assays were analyzed using a generalized linear model with repeated measures to compare the three matrices. Compound symmetry was used as the correlation matrix of measurements within virus. If the three matrices were significantly different, pairwise comparisons were performed. Only the Simplexa data had a normal distribution. For all the other assays a Box-Cox transformation was performed before the analyses. Since the Alere data was binary, a Chi-square test was used to compare the three matrices.

### Assessment of SRS in the Simplexa, Liat, and Alere Assays

Ten-fold serial dilutions of each of the five viruses were made in SRS containing the midpoint concentration of each component and tested in the Simplexa, Liat, and Alere assays in triplicate.

### Reactivity levels with infected cells

Infected cell stocks for influenza A H1N1pdm and influenza B Victoria-like were thawed and ten-fold serial dilutions prepared in PBS. For each dilution 50 μl was tested (in triplicate) with Sofia, Veritor, Simplexa, and CDC assays following the same protocol as described above.

### Comparing effect of SRS on cell-free virus and infected cells

Dilutions were selected for which the number of viral RNA copies should be about equal for infected cells and cell-free viruses (S4 Table). On the day of testing the appropriate replicate dilutions were prepared in SRS with infected cells and with cell-free virus. The order of each sample to be tested was randomized for each assay and tested in the Sofia, Veritor, Liat, and Simplexa assays.

### Dot blot assay

The Sofia and Veritor assays use antibodies to detect the nucleoprotein (NP) of influenza virus. We suspected that the IgG that had been used was able to interfere with the signal in these assays because it contained anti-NP antibodies that were competing with the detection antibodies. To determine if the IgG contained anti-NP antibodies we developed a dot blot assay which used influenza A NP protein spotted onto a polyvinylidene difluoride (PVDF) membrane. After preliminary experiments showed that the IgG from human serum did produce signal in the dot blot assay, we tested for the presence of anti-influenza NP IgG antibody in the 20 samples used for the quantification of respiratory sample components. PVDF membranes were spotted with 10 μl of 0.05 mg/ml of purified NP from influenza A/California/07/2009(H1N1pdm) (Model # 40205-V08B, Sino Biological, North Wales, PA, USA). After blocking for 1 h with agitation at room temperature membranes were ready. Serum IgG (1 mg/ml) diluted 1:250 was used as a positive control. Samples were diluted 1:10 in blocking buffer [1×PBS Tween 20 (KPL, Gaithersburg, MD, USA) with 5% Non-fat Milk] and incubated with the membrane at 4°C for about 17 h. Membranes were washed 5 times (1×PBS Tween 20) with agitation for 5 min. Membranes were incubated with chicken anti-human IgG HRP (Thermo Fisher Scientific) diluted blocking buffer, for 45 min at room temperature with agitation. Membranes were washed 5 times. Membranes were incubated for 10 min in 1-Step Ultra TMB Blotting Reagent (Thermo Fisher Scientific) and then washed for 10 min in deionized water to stop development. Dot intensity was determined using Gel Analyzer 2010a (http://www.gelanalyzer.com/) software with valley-to-valley background subtraction.

To rule out non-specific binding a monoclonal human IgG antibody (Model # HCA228, AbD Serotec, Raleigh, NC, USA) was purchased and a dot blot was performed using either blocking buffer alone, blocking buffer with monoclonal IgG (0.5 mg/ml) diluted 1:125, or blocking buffer with IgG from human serum (1.0 mg/ml) diluted 1:250 as the primary antibody.

In a separate experiment a 1:10 dilution of H1N1pdm virus was prepared in PBS, monoclonal IgG (0.5 mg/ml), and IgG from human serum (0.5 mg/ml) and 50 μl aliquots were prepared. Also, fresh midturbinate swabs were self-collected by a lab volunteer. All three sample types were tested with a clean swab and the virus in PBS was tested with the fresh midturbinate swabs in the Sofia and Veritor assays.

## Results

### Verification of respiratory sample components in NPS specimens

We had originally intended to use banked frozen NPS samples that had been collected in 3 ml of M4 transport media (Remel) and stored at ˗80°C for about four years for the quantification of respiratory sample components. However, preliminary testing showed that the measured OD_450_ index (sample OD/negative control OD) for IgG was significantly lower in these older samples than in specimens which had been collected in 1.5 ml of M6 transport media and stored at ˗80°C for less than 6 months (for n = 10; fresh mean OD_450_ index of 31.9 vs. old mean OD_450_ index of 8.1; p<0.001 for unpaired t-Test). Therefore, we collected and tested specimens that were less than six months old. The results of measuring the quantity of IgG, IgM, IgA, albumin, nucleic acid, and relative amount of anti-influenza NP IgG are shown in Table 2. The range of quantities measured for IgG, IgM, IgA, and albumin overlapped with the ranges for these constituents as used in the component effects testing (Table 1). Based on the statistical analysis there was no significant difference in concentrations between children and adults although several children did have exceptionally high amounts of albumin. These data confirm that the compositional makeup of materials collected by nasal/NP swab and diluted in transport media is consistent with previous reports respiratory secretions collected and measured directly assuming a volume of 10 to 50 μl per swab.

**Table 2:** Per swab quantities of respiratory sample components in NPS samples from children and adults.

		Children			Adults		
Target	Units	Min	Max	Median	Min	Max	Median
IgG	ug	2.20	38.64	9.50	2.69	24.78	5.91
IgM	ug	0.67	14.24	3.17	2.22	8.51	3.93
IgA	ug	0.92	49.02	4.43	3.46	47.17	7.49
Albumin	ug	4.79	527.35	94.67	10.05	46.56	24.57
Nucleic Acid	ug	1.46	24.10	8.10	3.51	31.50	8.01
Anti-NP IgG^a^	log volume	1.58	3.23	2.60	2.21	3.36	2.70

^a^ Determined by nucleoprotein dot blot.

Our results indicate that for all of the components there is no significant difference in quantity between children and adults, which is reflected in there generally being a poor correlation between component quantity and age (S5 Table). Multiple significant correlations were observed between the quantities of respiratory sample components. However, only three of the component combinations were significant in both groups.

### Effects of respiratory sample components on influenza assays

The results of the fractional factorial design testing showed minimal effects for most of the components tested across all of the assays. Surprisingly a strong negative effect of IgG was observed on the Sofia and Veritor assay signals (>80% reduction) with each of the viruses tested (S1 Fig). Results of the statistical analysis are shown in Tables 3 & 4. Using a linear model in which each of the components and the viruses was considered a variable, IgG had a significant negative effect on the CDC, culture, Sofia, and Veritor assays. While the effect was statistically significant for the CDC assay it was still functionally minimal with only a 0.24 Ct change. The effect on virus culture was also minimal with a 0.31–0.37 TCID_50_ change.

**Table 3:** Mean effect and p-values for the fractional factorial testing for the RATs.

	Veritor			Sofia		
Component	Effect	p-value	RE p-value^a^	Effect	p-value	RE p-value
Na+, K+	0.17	0.462	0.227	0.21	0.554	0.023 *
Ca++	0.29	0.223	0.050 *	0.02	0.962	0.849
Cells	-0.31	0.183	0.033 *	-0.71	0.052	2e-08 ***
Albumin	-0.31	0.190	0.035 *	-0.10	0.781	0.266
IgG	-4.27	< 2e-16 ***	0.001 **	-4.87	3.95e-14 ***	0.006 **
IgM	0.32	0.175	0.030 *	0.03	0.937	0.750
Mucin	-0.25	0.277	0.078	0.25	0.483	0.008 **

^a^ Used a random effects model in which the IgG value was allowed to vary between viruses.

* p-value < 0.05

** p-value < 0.01

*** p-value < 0.001.

**Table 4:** Mean effect and p-values for the fractional factorial testing for the NAATs and virus culture.

	CDC		Simplexa		Culture Day 2		Culture Day 3	
Component	Effect	p-value	Effect	p-value	Effect	p-value	Effect	p-value
Na+, K+	0.02	0.852	-0.25	0.089	-0.27	0.071	-0.24	0.066
Ca++	-0.09	0.415	0.13	0.387	0.15	0.311	0.18	0.172
Cells	-0.02	0.841	0.02	0.909	0.12	0.410	-0.08	0.526
Albumin	0.08	0.443	-0.09	0.522	-0.08	0.596	0.00	0.989
IgG	-0.24	0.026 *	-0.20	0.183	-0.37	0.015 *	-0.31	0.022 *
IgM	-0.15	0.148	0.00	0.982	0.05	0.701	0.09	0.470
Mucin	0.04	0.700	-0.15	0.307	-0.01	0.942	-0.02	0.895

* p-value < 0.05

The data for Sofia and Veritor assays were also analyzed with a random effects model where the IgG effect was allowed to vary across viruses (Table 3). This decreased the standard error and increased the significance for the remaining components. With the random effects model the Ca^2+^ and IgM had a significant positive effect on the Veritor assay while the A549 cells and albumin had a negative effect. Also, with the Sofia assay, the Na^+^ and K^+^ ions and mucin had a significant positive effect and the A549 cells had a significant negative effect. These last effects were relatively small.

With the exception of strong suppression by IgG of the antigen assay signals, the effects of the nasal secretion components for all of the assays, though significant in some cases, were relatively small. This being the case, we arbitrarily chose the midpoint concentrations of the literature ranges for our SRS formulation.

**Fig 1:**
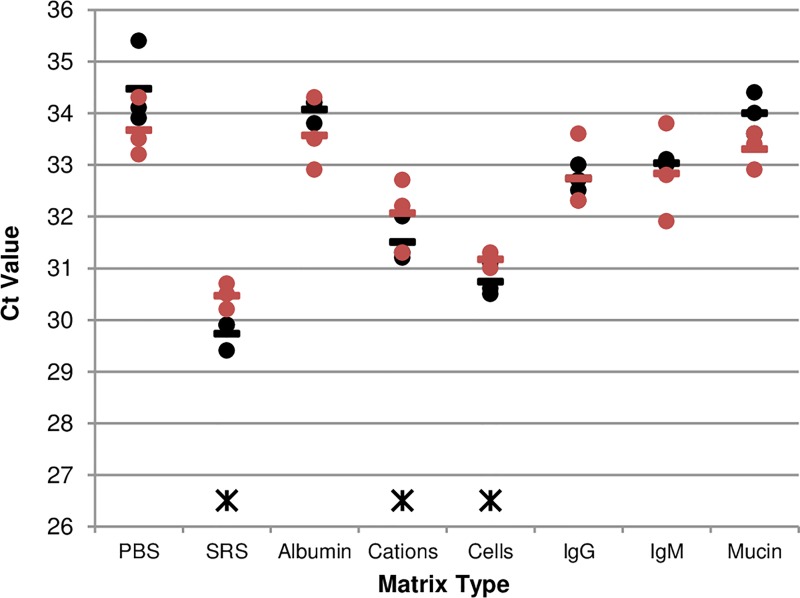
The results of testing each component’s effect on the Liat assay. H1N1pdm was diluted in each of the matrices containing each of the components and tested in triplicate in the Liat assay. The experiment was performed twice with the repeats shown in black and red. Each matrix was compared to PBS using the t-test. Bars indicate the mean of the three replicates for each repeat. Asterisks indicate a significant difference from PBS alone.

The results of testing the effects of sample components on the Liat assay showed a significant reduction of the Ct value was observed with SRS (p-value = 0.018), cations (p-value = 0.043), and cells (p-value = 0.021) when compared to PBS alone (~2 to 4 Ct units). (Fig 1 and S6 Table).

### Comparing SRS, PBS, and NCS matrices

**Fig 2:**
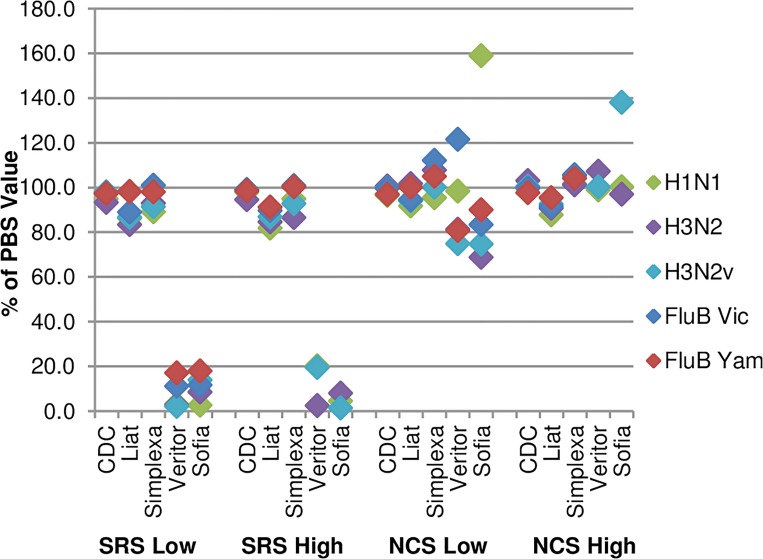
Results of testing virus spiked in PBS, SRS, and NCS with six assays. Ct and antigen assay signal results were normalized as a percentage of the PBS result value. Data for high and low concentrations of virus are shown.

Results from comparing the detection of the five influenza viruses in PBS, SRS, and NCS with the Sofia, Veritor, CDC, Simplexa, and Liat assays are shown in Fig 2 and Table 5. Assay signals for the five viruses in SRS or NCS are normalized as a percentage of the assay signal for the same viruses in PBS.

**Table 5:** P-values from statistical analysis of matrix comparison data.

	High Concentration			Low Concentration		
Assays	NCS vs. PBS	NCS vs. SRS	PBS vs. SRS	NCS vs. PBS	NCS vs. SRS	PBS vs. SRS
CDC	0.625	**0.019**	**0.0028**	0.083	**0.011**	**0.0013**
Liat Ct	**<0.001**	**0.0011**	**<0.001**	**0.017**	**0.0020**	**<0.001**
Simplexa	**0.0013**	**<0.001**	**0.011**	0.053	**<0.001**	**0.0046**
Sofia	0.072	**<0.001**	**<0.001**	0.635	**<0.001**	**<0.001**
Veritor	0.320	**0.0012**	**0.0013**	0.102	**<0.001**	**<0.001**

p-values <0.05 highlighted in bold.

For all of the NAAT assays (except Alere i), spiking virus into SRS resulted in a significant lowering of the Ct values observed, when compared to PBS. Pooling the results for all viruses tested, the average ΔCt differences for the Liat, Simplexa, and CDC assays were ˗4.1, ˗1.6, and ˗0.7 for high level of viruses and ˗4.2, ˗2.0, and ˗1.4 for low virus levels, respectively, a potential improvement in assay performance over PBS. The results using pooled NCS as a sample matrix are less consistent. There appears to be a trend toward lower Cts with the CDC and Liat assays, while the Simplexa assay Cts trended higher. Only the Liat ΔCts of -2.6 and -1.3 for high and low virus levels in pooled NCS were significant. No significant differences between the matrices (p-value = 0.141) was observed for the Alere i. Since the data from the Alere i assay were binary (positive or negative) and the sample size small, it would not have been possible to detect anything other than large negative effects on the assay.

For the RATs (Sofia and Veritor), SRS significantly reduced signal (>80% reduction) with a negative impact on apparent sensitivity. This is consistent with the previous observation that serum derived IgG (as a constituent of the SRS) suppresses the assay signal in the RATs. For the Sofia assay, the results of the pooled NCS with low levels of virus showed signal reduction for four of the five viruses. H1N1pdm displayed unusually high signal values in the NCS, but when this virus’ data was omitted from the matrix comparison, NCS produced a significantly lower value than PBS (p-value = 0.0040). No reduction in signal was observed with the high levels of virus.

### Performance of Alere, Liat, and Simplexa assays with SRS matrix

We compared the performance of three NAATs with viruses in SRS. All three assays performed similarly for influenza A while some assay to assay variation was observed with influenza B (Table 6).

**Table 6:** Serial dilutions of viruses in SRS in the Simplexa, Liat, and Alere assays.

	H1N1pdm			H3N2			H3N2v			B Vic			B Yam		
Dilution	L^a^	A	S	L	A	S	L	A	S	L	A	S	L	A	S
-2	-^b^	-	-	-	-	-	-	-	-	-	-	-	-	**2**	-
-3	-	-	**3**	-	**3**	**3**	-	**3**	**3**	-	-	**3**	**3**	**1**	**3**
-4	**3**	**3**	**3**	**3**	**3**	**3**	**3**	**3**	**3**	**3**	**3**	**1**	**3**	0	**3**
-5	**2**	**1**	**1**	**3**	**3**	0	**3**	0	**3**	**3**	**3**	0	**3**	0	0
-6	0	0	0	0	0	0	0	0	0	**1**	**1**	0	0	0	0
-7	0	0	-	0	-	-	0	-	-	0	0	-	-	-	-

^a^ L = Liat, A = Alere, S = Simplexa

^b^ Values shown are the number of positive replicates out of 3. A dash indicates the dilution was not tested in the assay.

### Sensitivity of the assays with infected cells

Infected cells are a component of clinical specimens which contain viral proteins and RNA (targets of diagnostic assays). Table 7 demonstrates that the Sofia and Veritor assays detected approximately 24 H1N1pdm infected cells and 240 influenza B (Victoria-like) infected cells. The molecular tests (CDC, Simplexa) were about 1 log more sensitive. Using the in-house quantitative RT-PCR the calculated RNA copies per infected cell were 45,000 viral RNA copies/cell (H1N1pdm), 17,000 viral RNA copies/cell (influenza B, Victoria-like), 9 RNaseP copies/cell (infected cell lines), and 29 RNaseP copies/cell (uninfected cells). This suggests that 1–2 cells lysed in VTM could be sufficient for virus detection by molecular methods and infected cells are a rich source of diagnostic assay targets.

**Table 7:** Results of testing 10-fold serial dilutions of infected cells with each assay.

	H1N1pdm				Flu B Victoria			
Cells	Sofia	Veritor	CDC	Simplexa	Sofia	Veritor	CDC	Simplexa
2400	-	-	-	-	**3**	**3**	**3**	**3**
240	**3**	**3**	**3**	**3**	**3**	**3**	**3**	**3**
24	**3**	**1**	**3**	**3**	0	0	**3**	**3**
2.4	0	0	**3**	**3**	0	0	**3**	**1**
0.24	0	0	0	**1**	0	0	0	0

### Infected cells versus cell free virus with PBS and SRS

To normalize the differences between virus and infected cells in PBS, the SRS values were converted to a percentage of the PBS value (Table 8 and S2 Fig). For the Sofia and Veritor assays, the viruses and infected cells in SRS produced about 26% or lower signal than in PBS. With the Liat assay lower Ct values were found in SRS (ΔCt ~ 2 to 4). But, for the Simplexa assay the results were somewhat mixed. In general, these results were consistent with the previous matrix comparison results that were conducted with only virus thereby suggesting that the detection of target molecules in virus and infected cells are affected in the same manner by the SRS matrix.

**Table 8:** Comparing the effect of SRS versus PBS on the detection of infected cells or cell free virus (analytes). (SRS Value/ PBS value).

	H1N1		B Victoria	
Assay	Virus	Cells	Virus	Cells
Sofia	3.7%	3.1%	15.8%	15.7%
Veritor	26.2%	9.4%	7.1%	20.5%
Simplexa	90.3%	102.0%	98.4%	96.7%
Liat	84.1%	93.9%	92.9%	86.3%

### Characterization of IgG suppression of antigen assay signals

Table 2 summarizes the quantitative dot measurements of the samples analyzed in this method. All samples were positive and a strong correlation between dot intensity and IgG concentration was observed (S5 Table).

**Fig 3:**
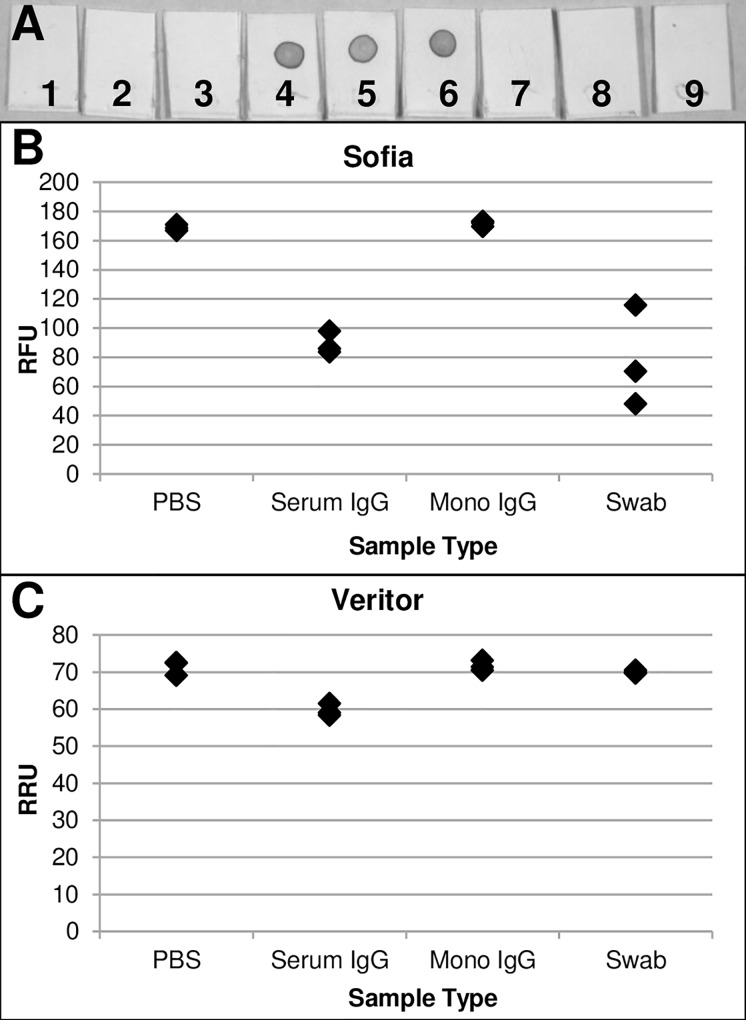
(A) Dot blot for the detection of IgG antibodies specific to the H1N1pdm NP. Membranes 1 to 3 were negative controls, 4 to 6 were human serum IgG, and 7 to 9 were monoclonal IgG. (B-C) Comparison of PBS, serum IgG, monoclonal IgG, and respiratory swabs with the Sofia and Veritor assays. H1N1pdm was diluted 10-fold from stock in PBS, serum IgG, or monoclonal IgG, absorbed on a clean swab, and tested. Also, a set of samples with virus diluted in PBS was absorbed on a freshly collected NPS and tested.

To provide further evidence that the dot blot assay was detecting anti-NP antibodies and was not just a result of any IgG being detected, we tested a monoclonal IgG antibody in the assay. In Fig 3A membranes 4 to 6 show strong reactivity of human IgG from serum with the spotted nucleoprotein while membranes 6 to 9 were unreactive with just the monoclonal human IgG.

To show that the suppression of the Sofia and Veritor assays was not just caused by the presence of any IgG, we also tested the monoclonal IgG with these assays. Again, human serum IgG suppressed the assay signal in the Sofia and Veritor assays, but the monoclonal IgG had no effect (Fig 3B and 3C). The effect with serum IgG was less than what was observed in previous testing due to the lower concentration used in this experiment (0.45 mg/ml versus 0.71 mg/ml). To see if a real clinical sample could also affect these assays, freshly collected midturbinate swabs were tested. The swabs had a negative effect on the Sofia assay, but not the Veritor assay. As both assays were inhibited by higher concentrations of antibody in previous experiments this observation could reflect relative sensitivity to the level of the interferant. Characterizing the levels and affinities of the anti-NP antibodies would be needed to determine if this is a real difference. Also, in this testing we estimate the contents of the swabs were diluted about five-fold when added to the 50 μl of sample. Since the inhibitory effect is concentration dependent, it is likely that a lower volume of sample would have resulted in a greater observed effect.

## Discussion

The findings of this study support using a simulated respiratory sample matrix for influenza test development and validation activities as initially called for by the WHO Global Influenza Programme [9]. Leftover human clinical respiratory specimens can be pooled for limit of detection and other analytical studies according to Food and Drug Administration (FDA) guidance [10,11]. While pooled samples are often used for analytical studies, such samples have limited volumes, vary constitutively, and may be altered by storage or handling. Hence findings from studies with pooled samples may not repeat with a different pool, precluding comparability across studies. The lower levels of IgG in older stored samples, compared to recent specimens observed during our study highlight the potential variability of pooled sample composition. FDA guidance also suggests viral transport medium or a simulated matrix can be used for influenza testing if analytical performance of an assay is equivalent in simulated and natural clinical matrices. Thus, SRS spiked with influenza virus could conceivably parallel pooled influenza-negative clinical specimens for influenza virus detection in analytical experiments and evaluations, enable side-by-side assessments of assay differences, and facilitate earlier optimizations when emerging virus isolates are first available and clinical samples are restricted or rare.

Ideally, a simulated matrix would be applicable to all methods or platforms, and enable a standardized means to assess analytical accuracy, especially with novel and newly emerging viruses. Such an approach could enable developers to more quickly assess analytical accuracy of existing tests, validate changes as needed, and also facilitate analytical assessments with new influenza tests. Notably, a simulated saliva matrix spiked with virus is a precedent recognized for standardized testing of decontamination procedures for materials challenged with biological aerosols [12].

The SRS formulation we used incorporates the mid-point concentrations of major ionic, protein, nucleic acid and cellular components documented in cited literature reports. A small sampling of clinical archived samples verified the applicability of the literature-reported ranges. Importantly, the relative component levels in samples from children and adults were similar. No previous reports were found that documented comparability of respiratory secretions from children and adults. This potentially justifies the use of a single SRS formulation to represent samples from both groups.

With the NAAT assays, SRS and pooled NCS performed differently than PBS even though minimal effects were observed for the individual components in the fractional factorial experiment. Differences in the concentrations of the components used in these experiments likely account for this discrepancy. The significant positive effect of cations and cells with the Liat assay were similar in magnitude to SRS and suggested improved extraction efficiency by a carrier effect from cellular nucleic acid, especially with low viral RNA copy numbers [13,14]. Pooled NCS was shown to have a similar but less pronounced effect.

In contrast, findings with the two rapid antigen tests with SRS, showed significantly reduced signal compared to spiking in PBS. The strong suppression of the RAT signal values by IgG identified in the fractional factorial experiment anticipated this finding. Even though the IgG used had been purified from the serum of normal human donors, the majority of IgG present in the respiratory secretions of humans and other animals comes from serum [15–17], which suggests serum IgG should be representative of the IgG in human respiratory secretions. Suppression with multiple lots of commercially obtained serum-derived IgG, anti-NP reactivity in all 20 nasal swab samples tested, correlation of the intensity of the dot blot reactivity with total IgG in the sample, and no observed signal suppression with an irrelevant human monoclonal IgG indicate that signal suppression is due to specific anti-influenza NP antibodies. These antibodies were even detected in individuals less than one month of age who were unlikely to have been previously infected with influenza yet may have maternal pathogen specific antibodies transferred to the fetus through the placenta and to infants via breast milk [18–20]. The development of host anti-NP antibodies was previously described following viral infection or vaccination in humans and other animals [21,22]. Repeated exposure (infection or vaccination) to this highly conserved antigen, ideal for these broadly reactive immunoassays, is likely to result in boosting and maturation of the antibody response as described for HA antibodies [23]. Additionally, a previous study from this lab demonstrated that RAT reactivity was directly correlated to the amount of NP in virus isolates [24]. Therefore, blocking of NP epitopes targeted by the RAT assay monoclonal antibodies or clearing of host antibody NP aggregates are possible mechanisms by which anti-NP antibodies in respiratory secretions may be an unrecognized factor in reducing clinical performance of RATs compared to other assay formats [25,26].

While the pooled NCS in the matrix comparison did not have a large effect on the RATs these results should be viewed with caution. Even though the SRS had been designed to directly mimic a respiratory secretion, the respiratory secretion for the pooled NCS had been diluted >60-fold when the original swab was collected and placed in transport media resulting in much lower concentrations of sample components than if the secretion had been used directly. Since IgG affects these assays in a concentration dependent manner the concentration in the pooled NCS in transport media was generally too low to have a significant effect. These results clearly demonstrate that analytical data determined using NCS in transport media for development of direct swab assays may falsely overestimate sensitivity, which highlights another advantage of SRS in that it is applicable to assay designs using a direct swab specimen as well as those designs requiring use of transport media.

These findings represent a challenge to rapid antigen test manufacturers. While all of the patient nasal samples tested demonstrated some level of anti-NP thereby suggesting a wide prevalence in the population, the distribution, levels, specificities and affinities of these antibodies are not known. The prevalence profile of anti-NP antibodies could be dynamic, as annual vaccination and infections rates vary across populations. Additional characterization of anti-NP IgG levels and reactivity in nasal secretions and their impact is needed to better define an SRS formulation representative of clinical specimens for use with RAT assays.

Our study did confirm that infected cells are a rich source of assay targets (both viral RNA and NP) for NAAT and RAT tests with 2.4 and 24 cells detected respectively. Though the infected cell preps were extensively washed, we were unable to rule out contributions from “free” RNAs, proteins, or released virus in the infected cell preps. Understanding the contributions of free virus or cell-associated components remains important for optimizing collection of clinical specimens. Rapid antigen tests may be quite suitable for detecting virus when it is actively replicating and a cell-enriched quality specimen is obtained [27]. Notably, analytical testing could be more representative for both nucleic acid amplification and rapid antigen tests by adapting an SRS to include infected cells.

The fractional factorial design used was limited to the major components of nasal secretions and only looked at major effects. Additionally, all of the single factor effects could be confounded with two factor interactions. Further analysis of some of the small, but significant effects observed and expansion to capture secondary effects could improve recognition of suppressing or enhancing effects with assay chemistries. The components studied represent approximately 90% of the biological and chemical constituents documented in respiratory secretions. Analyses of the remaining components could reveal additional effects on assay performance. IgA was not included in the SRS formulations for cost reasons, but it is possible that like IgG it could also have an effect on assay performance.

The result of our study demonstrate significant effects from components in respiratory samples that can impact analytical findings from different influenza virus detection methods thereby highlighting the importance of understanding interactions in clinical samples and spiked virus concentrations with analytical experiments. While the formulated SRS matrix appears to mimic the effects of clinical samples for detecting influenza virus with nucleic acid amplification assays, further work is needed to characterize the extent of signal suppression inherent in virus positive clinical samples with NP antigen based tests. The identification of anti-NP antibodies as a potential interference in the RAT assays requires caution when inferring clinical performance from analytical studies using leftover clinical samples as a matrix.

Regardless of the method used, an accurate clinical diagnosis depends heavily on the quality of the specimens collected and prepared. If samples are not collected from anatomical sites where the virus is replicating and shed, or if samples are not handled, stored, and transported appropriately, false‐negative tests may ensue irrespective of the analytical validity of any particular assay method. Either the current SRS or further refined SRS formulations would allow for the production of a cost effective matrix that could overcome these issues and improve the validity of analytical testing.

## Supporting Information

S1 Fig Results of fractional factorial design SRS testing. These charts show the estimated effect of each of the SRS components on the detection of each virus in each assay.
(TIF)

S2 Fig Comparison of the effect of SRS on virus versus infected cells. H1N1pdm and influenza B Victoria-like virus were diluted in PBS and SRS and tested in the Sofia, Veritor, Simplexa, and Liat assays.
(TIF)

S1 Table Log_10_ dilutions used for the SRS component testing. (DOCX)

S2 Table Simulated respiratory secretion formulations. (DOCX)

S3 Table Log_10_ dilutions from stock used for comparing SRS, PBS, and NCS matrices. (DOCX)

S4 Table Log_10_ dilutions from stock used for testing infected cells and cell-free virus. (DOCX)

S5 Table Significant correlations between respiratory sample components. (DOCX)

S6 Table Effects of SRS components on H1N1pdm in the Liat assay. (DOCX)
