# Supplementary figures and images for: Simulated Respiratory Secretion for Use in the Development of Influenza Diagnostic Assays
Source: PLoS One. 2016 Nov 21;11(11):e0166800. doi: 10.1371/journal.pone.0166800 (PMC5117718; doi:10.1371/journal.pone.0166800)

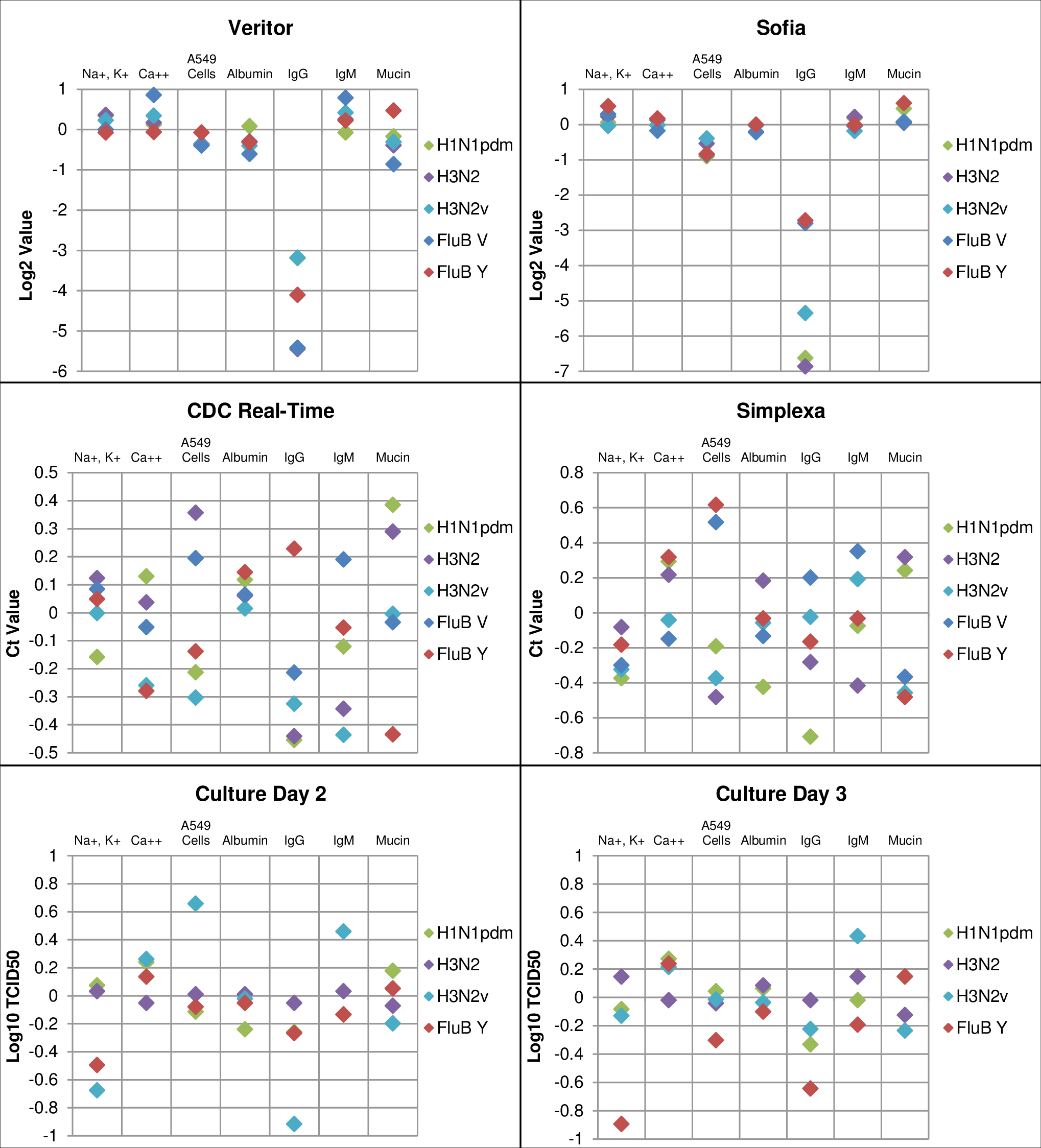

Supplement: S1 Fig — These charts show the estimated effect of each of the SRS components on the detection of each virus in each assay. (TIF) [file pone.0166800.s001.tif]

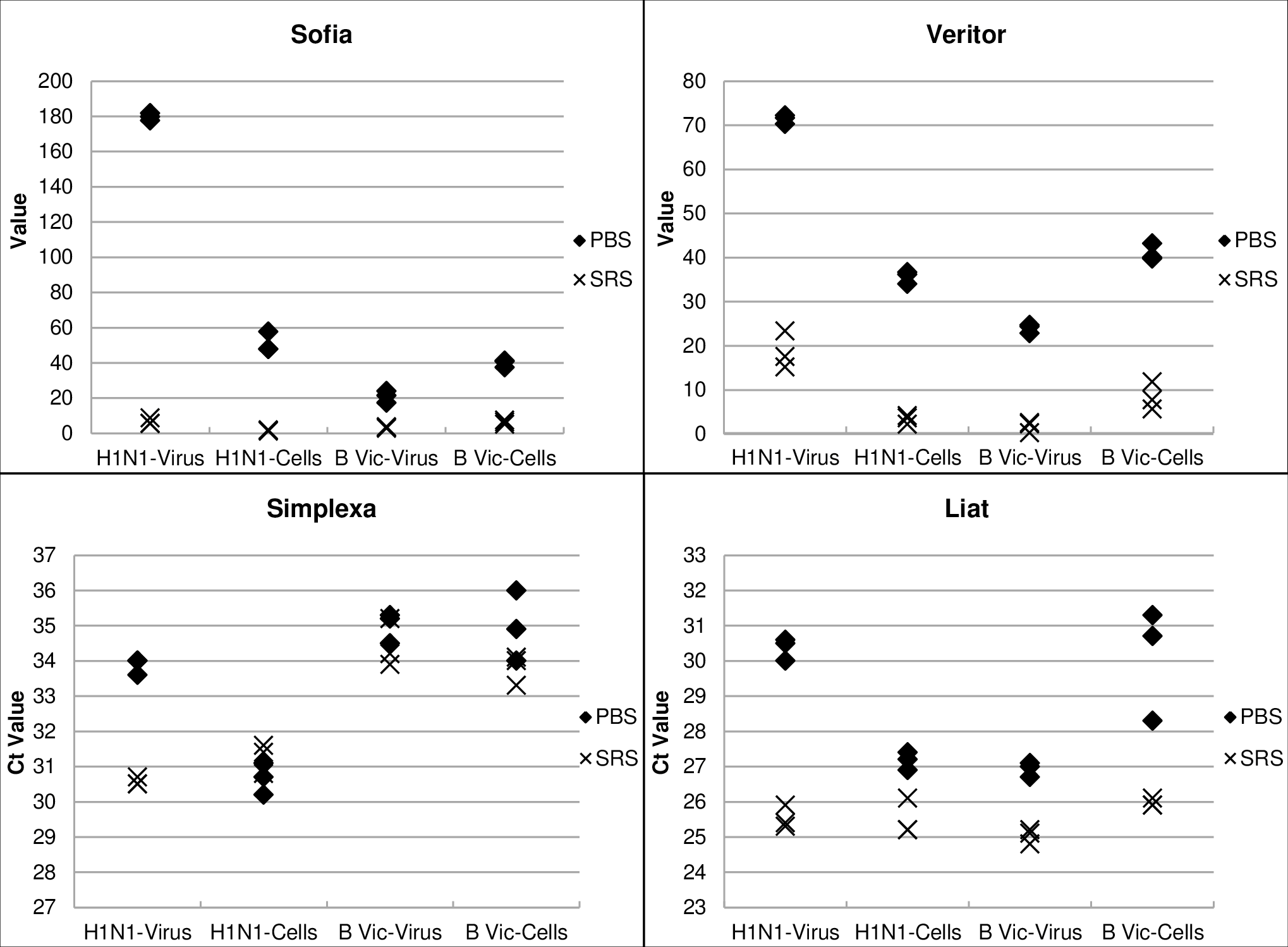

Supplement: S2 Fig — H1N1pdm and influenza B Victoria-like virus were diluted in PBS and SRS and tested in the Sofia, Veritor, Simplexa, and Liat assays. (TIF) [file pone.0166800.s002.tif]
