# Supplementary material for: Simulated Respiratory Secretion for Use in the Development of Influenza Diagnostic Assays
Source: PLoS One. 2016 Nov 21;11(11):e0166800. doi: 10.1371/journal.pone.0166800 (PMC5117718; doi:10.1371/journal.pone.0166800)
Supplement: S1 Table — (DOCX) [file pone.0166800.s003.docx]

| S1 Table. Log_10_ dilutions used for the SRS component testing | | | | | |
| --- | --- | --- | --- | --- | --- |
|  | Veritor | Sofia | CDC | Simplexa | Culture |
| H1N1pdm | -1 | -1.5 | -3 | -3 | -1 |
| H3N2 | -1 | -1 | -2.5 | -3 | -1 |
| H3N2v | -1 | -1 | -3 | -3 | -1.5 |
| B Victoria | -1 | -1 | -3.5 | -2.5 | -1 |
| B Yamagata | -1 | -1 | -4 | -3 | -1.5 |
