# Supplementary material for: Simulated Respiratory Secretion for Use in the Development of Influenza Diagnostic Assays
Source: PLoS One. 2016 Nov 21;11(11):e0166800. doi: 10.1371/journal.pone.0166800 (PMC5117718; doi:10.1371/journal.pone.0166800)
Supplement: S2 Table — (DOCX) [file pone.0166800.s004.docx]

| S2 Table. Simulated respiratory secretion formulations | | | | | | | |
| --- | --- | --- | --- | --- | --- | --- | --- |
| Formulation | Na+, K+ | Ca++ | A549 Cells | Albumin | IgG | IgM | Mucin |
| 1 | + | + | + | - | - | - | + |
| 2 | - | + | + | + | + | - | - |
| 3 | + | - | + | + | - | + | - |
| 4 | - | - | + | - | + | + | + |
| 5 | + | + | - | - | + | + | - |
| 6 | - | + | - | + | - | + | + |
| 7 | + | - | - | + | + | - | + |
| 8 | - | - | - | - | - | - | - |
