# Supplementary material for: Simulated Respiratory Secretion for Use in the Development of Influenza Diagnostic Assays
Source: PLoS One. 2016 Nov 21;11(11):e0166800. doi: 10.1371/journal.pone.0166800 (PMC5117718; doi:10.1371/journal.pone.0166800)
Supplement: S3 Table — (DOCX) [file pone.0166800.s005.docx]

| S3 Table. Log_10_ dilutions from stock used for comparing SRS, PBS, and NCS matrices | | | | | | |
| --- | --- | --- | --- | --- | --- | --- |
|  | Veritor | Sofia | CDC | Simplexa | Alere | Liat |
| H1N1pdm | -1, -2 | -1, -2 | -2, -3 | -2, -3 | -4, -5 | -3, -4 |
| H3N2 | -1, -2 | -1, -2 | -2, -3 | -2, -3 | -3, -4 | -3, -5 |
| H3N2v | -1, -2 | -1, -2 | -2, -3 | -2, -3 | -3, -4 | -3, -4 |
| B Victoria | -1 | -1 | -2.5, -3.5 | -1.5, -2.5 | -5, -6 | -3.5, -5 |
| B Yamagata | -1 | -1 | -3, -4 | -2, -3 | -2, -3 | -4, -5 |
