# Supplementary material for: Simulated Respiratory Secretion for Use in the Development of Influenza Diagnostic Assays
Source: PLoS One. 2016 Nov 21;11(11):e0166800. doi: 10.1371/journal.pone.0166800 (PMC5117718; doi:10.1371/journal.pone.0166800)
Supplement: S4 Table — (DOCX) [file pone.0166800.s006.docx]

| S4 Table. Log_10_ dilutions from stock used for testing infected cells and cell-free virus | | | | |
| --- | --- | --- | --- | --- |
| Assay | H1N1 Virus | H1N1 Cells | FluB Vic Virus | FluB Vic Cells |
| Simplexa/Liat | -2.5 | -2.6 | -3.0 | -3.5 |
| Sofia/Veritor | -1.0 | -1.2 | -1.0 | -1.8 |
