# Supplementary material for: Simulated Respiratory Secretion for Use in the Development of Influenza Diagnostic Assays
Source: PLoS One. 2016 Nov 21;11(11):e0166800. doi: 10.1371/journal.pone.0166800 (PMC5117718; doi:10.1371/journal.pone.0166800)
Supplement: S5 Table — (DOCX) [file pone.0166800.s007.docx]

| S5 Table. Significant correlations between respiratory sample components | | | | |
| --- | --- | --- | --- | --- |
|  | Children | | Adults | |
| Components | rho | p-value | rho | p-value |
| IgG vs Dot Intensity | 0.950^a^ | <0.001^a^ | 0.818 | 0.0038 |
| IgG vs IgM | 0.685 | 0.029 | - | - |
| IgG vs Albumin | 0.806 | 0.0049 | 0.745 | 0.013 |
| IgG vs Nucleic Acid | 0.661 | 0.038 | - | - |
| IgM vs IgA | 0.733 | 0.016 | 0.733 | 0.016 |
| IgM vs Albumin | 0.709 | 0.022 | - | - |
| IgM vs Nucleic Acid | 0.745 | 0.013 | - | - |
| Albumin vs Nucleic Acid | 0.745 | 0.013 | - | - |
| ^a^ Result show with a single outlier removed. With the outlier rho = 0.624, p-value = 0.054.  - Since only significant correlations are shown in this table a dash is shown for comparisons in a particular age group that did not have a p-value above 0.05. | | | | |
