# Supplementary material for: Simulated Respiratory Secretion for Use in the Development of Influenza Diagnostic Assays
Source: PLoS One. 2016 Nov 21;11(11):e0166800. doi: 10.1371/journal.pone.0166800 (PMC5117718; doi:10.1371/journal.pone.0166800)
Supplement: S6 Table — (DOCX) [file pone.0166800.s008.docx]

| S6 Table. Effects of SRS components on H1N1pdm in the Liat assay | | | | |
| --- | --- | --- | --- | --- |
| Sample | Average Ct | Std Dev | % PBS | p-value |
| SRS | 29.73 | 0.29 | 86% | 0.00034 |
| Cations | 31.50 | 0.44 | 91% | 0.0026 |
| Cells | 30.73 | 0.32 | 89% | 0.00090 |
| Albumin | 34.07 | 0.23 | 99% | 0.23 |
| IgG | 32.73 | 0.25 | 95% | 0.012 |
| IgM | 33.03 | 0.06 | 96% | 0.019 |
| Mucin | 34.00 | 0.40 | 99% | 0.21 |
| PBS | 34.47 | 0.81 | 100% |  |
